# Analyzing the Azorubine (E122) Adsorption Mechanism on Alumina Using Molecular Dynamics Simulation and the Density Functional Theory Approach

**DOI:** 10.1002/open.70322

**Published:** 2026-10-20

**Authors:** Anouar Ameziane El Hassani, Malika Khnifira, Issam Ameziane El Hassani, Mohamed Adadi, Mohammed ALSaeedy, Adil Touimi Benjelloun, Mohamed Abdennouri

**Affiliations:** ^1^ Laboratory of Engineering, Modeling and Systems Analysis, LIMAS, Faculty of Sciences Dhar El Mahraz Sidi Mohammed Ben Abdellah University, USMBA Fez Morocco; ^2^ Engineering Laboratory of Organometallic Molecular, Materials and Environment (LIMOME), Faculty of Sciences Sidi Mohamed Ben Abdellah University, USMBA Fez Morocco; ^3^ Multidisciplinary Research and Innovation Laboratory, FP Khouribga Sultan Moulay Slimane University of Beni Mellal Khouribga Morocco; ^4^ Laboratory of Medicinal Chemistry, Faculty of Medicine and Pharmacy Mohammed V University in Rabat Rabat Morocco; ^5^ Laboratory of Engineering, Systems and Applications, National School of Applied Sciences Sidi Mohamed Ben Abdallah University Fez Morocco; ^6^ Faculty of Arts & Sciences, Department of Chemistry Kocaeli University Kocaeli Turkey

**Keywords:** adsorption process, alumina, azorubine (E122), density functional theory, molecular dynamics simulation

## Abstract

This study investigates the adsorption mechanisms of the organic azo dye azorubine (E122) on the surface of alumina (γ‐Al_2_O_3_), using molecular dynamics simulations, density functional theory (DFT), noncovalent interaction, and reduced density gradient analyses. The chemical reactivity of E122 was evaluated using DFT with the B3PW91 hybrid exchange–correlation functional and the LANL2DZ relativistic pseudopotential basis set. Speciation analysis showed that pH strongly influences the distribution of E122's ionic forms according to their pKa values, with forms 1, 2, and 3 predominating across the studied pH range, and the dye exhibiting strong affinity for the adsorbent surface in aqueous medium. DFT analysis identified key active sites and electronic properties governing dye–surface interactions and adsorbent affinity. Form 2 showed the highest adsorption energy (−917.107 kcal/mol), followed by forms 1 and 3 (−861.553 and −806.286 kcal/mol, respectively). These adsorption properties were further confirmed through density field and energy distribution analyses, while radial distribution function profiles, with all interatomic distances within 3.5 Å, confirmed a chemisorption‐driven mechanism. Overall, γ‐Al_2_O_3_ is an effective, low‐cost, recyclable, and eco‐friendly adsorbent for dye removal.

## Graphical abstract

Molecular dynamics and density functional theory simulations reveal that azorubine (E122) is strongly chemisorbed onto γ‐Al_2_O_3_ (radial distribution function distances <3.5 Å), with form 2 showing the highest adsorption energy (−917.107 kcal/mol). Noncovalent interactions (NCI)/reduced density gradient and HOMO–LUMO analyses identify the active sites and NCIs (van der Waals, π–π, electrostatic) driving adsorption, positioning γ‐Al_2_O_3_ as an efficient, low‐cost adsorbent for dye removal from water.

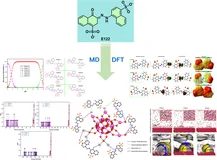

## 1 Introduction

The discharge of untreated or inadequately treated industrial wastewater is a major source of water pollution, particularly because of the presence of persistent organic contaminants such as synthetic dyes [1, 2, 3]. Among these pollutants, azo dyes are widely used in textile, food, pharmaceutical, and other industrial sectors and may pose significant risks to aquatic ecosystems and human health because of their chemical stability and potentially toxic degradation products [4, 5, 6, 7]. Consequently, the development of efficient, economical, and environmentally compatible methods for removing dyes from contaminated water remains an important environmental challenge.

Several technologies have been developed for dye removal from wastewater, including coagulation/flocculation, oxidation, membrane filtration, ion exchange, and adsorption [8, 9, 10]. Among these approaches, adsorption has attracted considerable attention because of its operational simplicity, relatively low cost, high removal efficiency, and versatility toward different classes of pollutants. The efficiency of adsorption strongly depends on the physicochemical properties of both the adsorbent and the adsorbate, as well as on the nature of the interactions established at the solid–liquid interface. Therefore, understanding the molecular mechanisms governing dye adsorption is essential for the rational design and optimization of efficient adsorbent materials [11, 12].

Metal oxides, particularly aluminum oxide (Al_2_O_3_), have been extensively investigated as adsorbents because of their surface reactivity, chemical stability, and favorable physicochemical properties [13, 14, 15]. Alumina can exist in several crystalline phases, including α‐Al_2_O_3_, γ‐Al_2_O_3_, θ‐Al_2_O_3_, and η‐Al_2_O_3_. Among these phases, γ‐Al_2_O_3_ is particularly attractive for adsorption applications because of its high specific surface area, porous structure, and abundance of surface active sites [16, 17, 18]. Previous experimental studies have reported the ability of alumina‐based materials to interact with various organic pollutants. However, the molecular‐level mechanisms controlling the interaction between specific dye molecules and γ‐Al_2_O_3_ surfaces remain insufficiently understood.

Azorubine (E122) is a synthetic azo dye widely used as a colorant and represents a relevant model pollutant for investigating the adsorption of anionic organic molecules. Although experimental studies have examined the removal of dyes using alumina and other metal oxides, a detailed molecular description of the adsorption process, including the preferred adsorption configurations, interaction sites, adsorption energies, and the dynamic behavior of the dye at the γ‐Al_2_O_3_/water interface, is still limited [19, 20]. In particular, experimental measurements alone do not provide direct access to the electronic interactions and atomistic mechanisms responsible for adsorption.

Computational approaches can provide complementary molecular‐level information that is difficult to obtain experimentally. Density functional theory (DFT) calculations are particularly useful for investigating the electronic structure of adsorbate–surface systems and evaluating the nature and strength of interactions between pollutant molecules and active surface sites [12, 19, 20]. Molecular dynamics (MD) simulations can further provide information on the structural and dynamic behavior of the adsorbate at the solid–liquid interface, including adsorption configurations, interfacial interactions, and the influence of the aqueous environment [21, 22, 23]. Monte Carlo (MC) simulations can additionally be employed to explore energetically favorable adsorption configurations and complement the molecular interpretation obtained from DFT and MD calculations.

Despite the growing application of computational methods to adsorption studies, a comprehensive molecular‐level investigation of azorubine adsorption on γ‐Al_2_O_3_ combining electronic structure calculations with molecular simulations remains limited. This constitutes the specific scientific gap addressed in the present work. Therefore, the objective of this study is to elucidate the adsorption mechanism of azorubine (E122) onto γ‐Al_2_O_3_ using a combination of DFT, MD, and MC simulations. DFT calculations were first employed to characterize the structural and electronic properties of the dye and to investigate its interactions with the alumina surface. MD simulations are then used to examine the behavior and stability of the adsorbate–adsorbent system in an aqueous environment, while MC simulations are employed to identify favorable adsorption configurations. The combined computational approach provides a molecular‐level understanding of the interactions governing E122 adsorption on γ‐Al_2_O_3_ and may contribute to the rational development of alumina‐based materials for dye removal from aqueous systems.

## 2 Computational Details

### 2.1 DFT Details

The software Gaussian 09 [16] was used for DFT analyses. Using hybrid functionals, such as hybrid correlation‐exchange functional and hybrid meta‐exchange–correlation functional, referred to as B3LYP, B3PW91, MPW1PW91, WB97XD, and M06‐2X, using the 6‐31G(d,p), 6‐311G(d,p) basis sets and the LANL2DZ basis set with a pseudopotential of the same name [17, 18, 19, 20], and preparing the structures for DFT calculations were made possible thanks in large part to this program. Understanding the electrical characteristics of the molecules is crucial for comprehending their behavior and interactions, and DFT research offers insight into these characteristics. The polarizable continuum model was used to investigate the effect of the implicit water solvent on binding energies. Software called Gaus View [12] was used to further analyze the DFT analysis results. This software provides an in‐depth understanding of the electronic structure and is a multipurpose wavefunction analyzer.

As per Koopmans’ theorem [21], the gap between HOMO and LUMO energies is responsible for the chemical reactivity of chemical compounds, which is ultimately determined by the global reactivity parameters. The characteristics and reactivity of the molecules are directly related to these quantities, such as electron affinity (*A*), ionization potential (*I*), chemical potential values (*μ*), chemical softness (*S*), chemical hardness (*η*), electronegativity (*χ*), and electrophilicity index (*ω*). These parameters are highly useful in explaining the reactivity patterns of the chemical systems and offer a conceptual grasp of the relationship between stability and structure [22, 23]. Furthermore, the partial charges of the studied molecules, the dipole moment, and the molecular electrostatic potential (MEP) map were calculated similarly. The interacting systems’ second‐order perturbation stability and electron‐transfer strength were understood through the use of the natural bond orbital (NBO) analysis; the interaction type and bond type were studied using the quantum theory of atoms in molecule analysis; and the active sites of the selected compounds were identified using Fukui indices. The parameters were computed using mathematical formulas found in the literature and are shown in Equations (1)–(8) [24, 25, 26].

Energy gap (Δ*E*_gap_):

(1) $$\Delta E_{\mathrm{gap}}=(E_{\text{LUMO}}-E_{\text{HOMO}})$$

Chemical softness (*η*):

(2) $$\eta =-\frac{1}{2}(E_{\text{HOMO}}-E_{\text{LUMO}})$$

Global hardness (*S*):

(3) $$S=\frac{-2}{\left(E_{\text{HOMO}}-E_{\text{LUMO}}\right)}$$

Global electrophilicity index (*ω*):

(4) $$\omega =\frac{\left(E_{\text{LUMO}}+E_{\text{HOMO}}\right)}{8}$$

Absolute electronegativity (*χ*):

(5) $$\chi =-\frac{1}{2}(E_{\text{LUMO}}+E_{\text{HOMO}})$$

Chemical potential (*µ*):

(6) $$µ=\frac{1}{2}(E_{\text{LUMO}}+E_{\text{HOMO}})$$

Electron charge transfer (Δ*N*):

(7) $$\Delta N=\frac{\chi_{\text{Alumina}}-\chi_{\mathrm{Dye}}}{2({\text{η}}_{\text{Alumina}}+{\text{η}}_{\mathrm{Dye}})}$$

Back donation energy (Δ*E*_b−d_):

(8) $$\Delta E_{b-d}=\frac{E_{\text{HOMO}}-E_{\text{LUMO}}}{8}$$

Furthermore, the binding energies between two atoms were calculated using second‐order perturbation theory. The stabilization energy *E* ^2^ [13] associated with the delocalization *i* → *j* is estimated for each donor (*i*) and acceptor (*j*) using Equation (8):

(9) $$E^{2}=\frac{q_{i}({F_{ij})}^{2}}{\left(E_{j}-E_{i}\right)}$$

where *q* *_i_* represents the «donor orbital» occupancy, *E* *_i_* and *E* *_j_* are diagonal elements, and *F* *_ij_* is off‐diagonal NBO Fock matrix elements.

### 2.2 MD and MC Simulations

In this study, MD was used to simulate the interaction of an **E122** dye with an alumina surface (Al_2_O_3_), using 10 Å unit cell parameters for an alumina crystal and focusing on the surface (113), which is a typical approach in materials science and physical chemistry to understand adsorption and surface phenomena at the atomic scale, using Material Studio software [27]. The substrate/adsorbate interface was modeled using a sequential MC and MD protocol within the BIOVIA Materials Studio 8.0 suite. First, the Adsorption Locator module was employed to conduct MC simulations to search for the most stable, lowest‐energy configurations of the adsorbate on the substrate. Subsequently, the optimal configuration identified by the MC search was used as the starting structure for the MD simulations, which were performed using the Forcite module to evaluate the dynamic equilibrium of the system. To replicate the experimental conditions, a MC simulation was performed with 150 water molecules (H_2_O) and a dye in a 42 × 42 × 42 Å^3^ cubic box with a vacuum layer of 42 Å on the *C*‐axis, a common configuration for simulating interfaces, thin films, or volume properties with periodic boundary conditions. During the MD simulations, the COMPASS force field was used to calculate the potential energy values required for molecular interactions [13, 28]. In addition, MD calculations were performed using five simulated annealing cycles at 298 K (20 000 steps per cycle) with a 4 × 4 × 2‐unit cell. The Ewald summation method and the atomic summation approach were used to adjust the electrostatic and van der Waals parameters using a cut‐off distance of 12.5. The NVT canonical set was executed using the Nose thermostat that maintains 298 K, with a short time step (1 fs) and long duration (1000 ps) to ensure that the system explores enough states and reaches thermodynamic equilibrium, thus collecting representative physical properties.

The interaction between **E122** dye and Al_2_O_3_ surface was estimated as the difference between the sum of the energy of the isolated molecule and isolated substrate (*E*_surface_ + *E*_molecule_) and the total energy (*E*_total_) of complex. Consequently, the adsorption energy was calculated using Equation (10) [29].

(10) $$E_{\text{ads}}=E_{\text{surface/}\mathrm{molecule}}-(E_{\text{surface}}+E_{\text{molecule}})$$

So, the binding energy (*E*_binding_) is the negative value of the adsorption energy, as follows:

(11) $$E_{\text{Binding}}=-E_{\mathrm{Ads}}$$

The radial distribution function (RDF) used to predict the distance between two atoms, *α* and *β*, is calculated by Equation (12) [29].

(12) $$g_{\alpha \beta }(r)=\frac{V}{N_{\alpha }N_{\beta }}\left(\sum_{i=1}^{N_{\alpha }}\frac{n_{i}\beta (r)}{4\pi r^{2}\mathrm{\Delta r}}\right)$$

where *N*, *n* *_i_* *β*(*r*), and *V* describe, respectively, the particle number, total number of *β* atoms in the spherical distance *r* of the *α* atom and simulation box volume.

### 2.3 Noncovalent Interaction (NCI)‐Reduced Density Gradient (RDG) Analysis

The NCI‐RDG analysis made it possible to evaluate the weak intermolecular interactions governing the three forms of azorubine and highlight their contribution to adsorption efficiency. This calculation was performed using the Multiwfn software [30]. These interactions were visualized and interpreted using the visual molecular dynamics interface, and quantitative color‐coded scatter plots for forms 1, 2, and 3 were subsequently generated using gnuplot [31].

## 3 The Choice of Method in Order to Better Describe the System Under Study

A study of the **E122** dye properties was carried out at the level of the DFT method with hybrid functionals of the type correlation‐exchange hybrid functional and meta‐exchange–correlation hybrid functional, called B3LYP, B3PW91, MPW1PW91, WB97XD, and M06‐2X, using the Pople basis set 6‐31G(d,p), 6‐311G(d,p), and the LANL2DZ basis set with a pseudopotential bearing the same name. Table 1 presents the optimized total energy values of the **E122** molecule obtained by the DFT method with the hybrid functionals (B3LYP, B3PW91, MPW1PW91, WB97XD, and M06‐2X) and Pople basis set (6‐31G(d,p), 6‐311G(d,p)) as well as the pseudopotential basis set LANL2DZ. Examination of the optimized total energy values shows that the azorubine molecule exhibits the lowest total energy value with the 6‐311G(d,p) basis set, followed by 6‐31G(d,p), with an important computation time, and the highest total energy value with the LANL2DZ pseudopotential basis set for all hybrid functionals, with a reasonable computation time. The energy gap Δ*E* values show that the azorubine molecule is weaker with the B3LYP and B3PW91 hybrid functionals than with the MPW1PW91, WB97XD, and M06‐2X hybrid functionals. However, the B3PW91 functional has a slightly smaller Δ*E* energy gap compared to that of the B3LYP functional. Indeed, the B3PW91 functional with the 6‐311G(d,p) basis set exhibits the highest value, followed by B3PW91 with the 6‐31G(d,p) basis set and then B3PW91 with the LANL2DZ basis set. Similar conclusions can be drawn from the overall softness σ results. In terms of reactivity, the B3PW91 hybrid functional with the LANL2DZ pseudopotential basis set best describes the system under study.

**TABLE 1:** Comparative analysis of certain chemical descriptors of the azorubine dye calculated in the ground state by the DFT method with different functionals (B3LYP, B3PW91, MPWAPW91, WB97XD, and M06‐2X) and different basis sets (6‐31G(d,p), 6‐311G(d,p), and LANL2DZ).

Functional Descriptor	B3LYP	B3PW91	MPW1PW91	WB97XD	M06‐2X
**DFT/functional/6‐31G(d,p)**					
*E*_Total_, u.a	−2201.774	−2201.148	−2201.466	−2201.290	−2200.169
*E*_g_, eV	2.903	2.897	3.294	6.532	5.024
*σ*, eV^−1^	0.689	0.690	0.607	0.306	0.398
**DFT/functional/6‐311G(d,p)**					
*E*_Total_, u.a	−2202.173	−2201.520	−2201.834	−2201.657	−2201.585
*E*_g_, eV	2.948	2.941	3.306	6.159	5.137
*σ*, eV^−1^	0.678	0.680	0.605	0.325	0.390
**DFT/functional/LANL2DZ**					
*E*_Total_, u.a	−1425.119	−1424.620	−1424.799	−1424.642	−1625.417
*E*_g_, eV	2.659	2.652	3.107	6.442	5.082
*σ*, eV^−1^	0.752	0.754	0.644	0.310	0.394

Consequently, the following section will be devoted solely to the presentation and analysis of the results of the E122 dye at the level of the DFT method with the B3PW91 hybrid functional and the LANL2DZ pseudopotential basis set.

## 4 Results and Discussion

### 4.1 Effect of pH on Reactivity of Azorubine (E122) Molecule

Pollutants’ solubility in water and pKa (acidity constant) are inextricably linked to their mobility, bioavailability, and fate in the aquatic environment (water and wet soils). While a pollutant's pKa influences whether it will be found in ionic or molecule form, affecting its persistence, toxicity, and interaction with organic or mineral substances in the soil, highly soluble pollutants or those whose ionized form is stable at a particular pH can spread more readily. Predictive research on the dye's solubility and pKa is carried out in this context [29]. This dye is regarded as soluble in aqueous media because of its polarization and ionization. Figure 1 displays the pKa values of several **E122** structures as a function of pH. As a function of pH, the distribution diagram displays the relative fraction (or percentage) of the basic and acidic forms. The distribution diagram shows which species predominates at various pH levels by displaying the relative proportion (in percentage) of the acidic and basic forms of an acid–base pair as a function of pH. The curves met at 50% when pH = pKa. In a highly acidic environment (pH 0–3), sulfonate groups remain in their anionic form because they are strong acids, while amine groups accept a proton to become cations, thus carrying a net positive charge. This ionic duality (anionic for sulfonates and cationic for amines) is fundamental to understanding the behavior of the molecule in acidic solution. Due to their extremely low pKa, Sun Yellow's sulfonate groups (─SO_3_^−^) are highly acidic and stay deprotonated (in ionic form) across the whole food pH range (pH 0–14), guaranteeing their solubility and stability in water, even in acidic or extremely basic situations. The **E122** molecule ionizes more in a very basic environment (pH > 10), frequently taking on a reddish‐orange or yellow tint, indicating its function as a colored pH indicator. It can be observed that other forms (4, 5, and 6) showed a low distribution at the pH range (almost nothing). Based on these results, we will study the three forms of E122 (1, 2, and 3) that were affected by pH.

**FIGURE 1:**
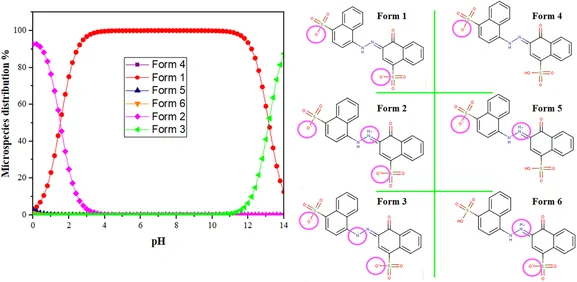
Microspecies distribution versus pH.

### 4.2 Molecular Geometry Analysis

The values of the structural parameters deemed relevant for the three forms of the azorubine molecule are reported in Figure 2 and Table 2.

**FIGURE 2:**
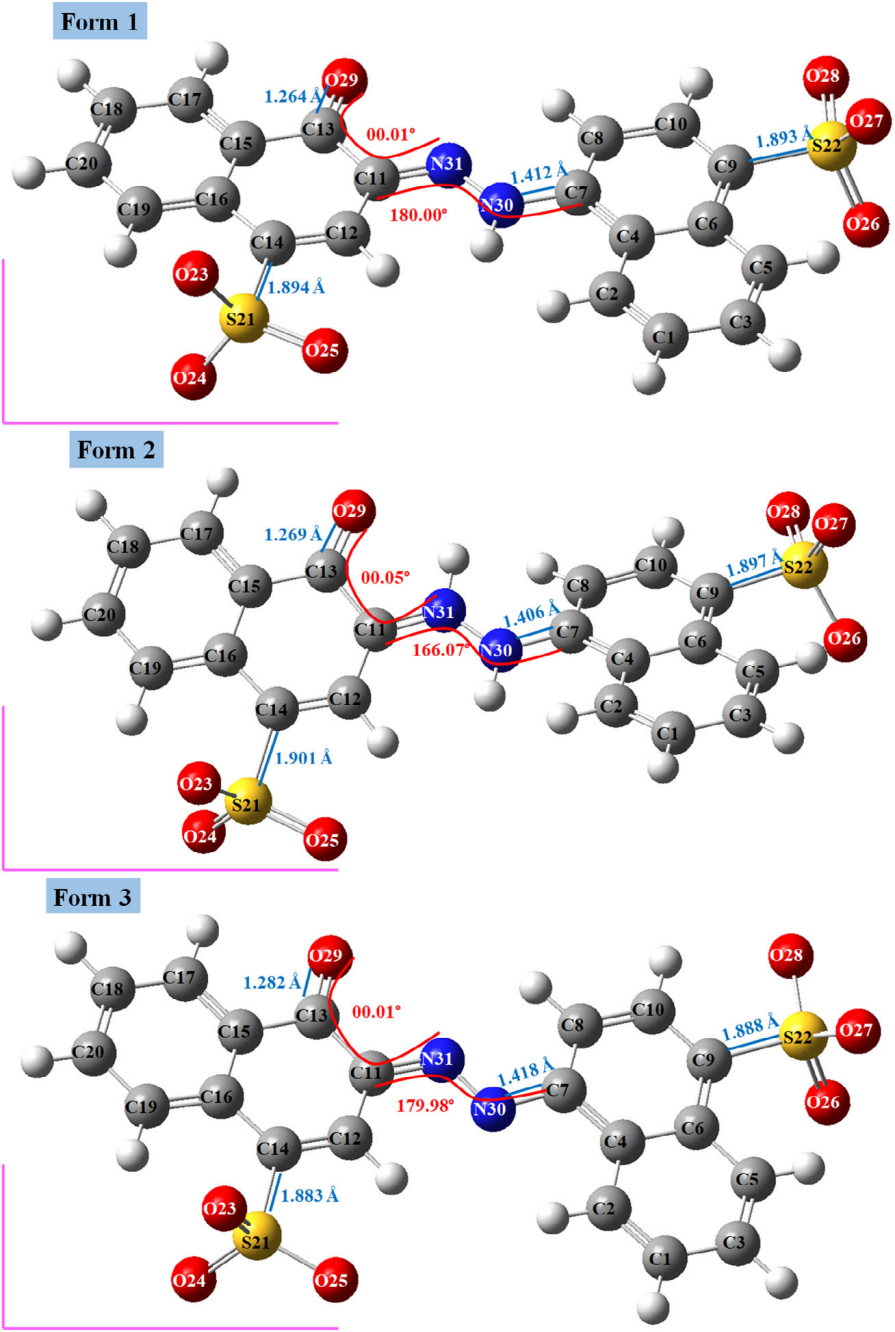
Optimized geometries of azorubine molecule shapes at the DFT/B3PW91/LANL2DZ.

**TABLE 2:** Bond lengths in (Å) of the studied dye forms, calculated at the DFT/B3PW91/LANL2DZ level.

Bond length, Å	Form 1	Form 2	Form 3	Bond length, Å	Form 1	Form 2	Form 3
C_9_–S_22_	1.893	1.897	1.888	S_21_–O_23_	1.641	1.636	1.650
C_14_–S_21_	1.894	1.901	1.883	S_21_–O_24_	1.641	1.637	1.650
C_11_–N_31_	1.336	1.341	1.378	S_21_–O_25_	1.641	1.635	1.641
N_31_–N_30_	1.333	1.347	1.315	S_22_–O_26_	1.643	1.635	1.645
N_30_–C_7_	1.412	1.406	1.418	S_22_–O_27_	1.643	1.640	1.647
C_13_–O_29_	1.264	1.269	1.282	S_22_–O_28_	1.640	1.639	1.647

The dihedral angle values [C_11_, N_31_, N_30_, and C_7_] for forms 1 and 3, both ≈180°, imply the planarity of both forms, a result of electron delocalization within the azorubine molecule. Similarly, the dihedral angle value [C_11_, N_31_, N_30_, and C_7_] for form 2 is ≈166.07°, indicating that this form is slightly twisted, likely due to the effect of protonation of nitrogen atom N31. In contrast, the dihedral angle [O_29_, C_13_, C_11_, and N_31_] for all three forms varies between 0.01° and 0.05°, demonstrating that the structures of the azorubine dye are not influenced by protonation and deprotonation.

It should also be noted that the average length of the single C─S bond in the three forms varied between 1.883 and 1.901 Å, which is significantly higher than the normal value for this bond (C─S = 1.82 Å) [32]; this could indicate the existence of an attractive‐repulsive electronic effect as well as their partial ionic character. The average values of the C_13_═O_29_ double bond in these three forms vary between (1.264 and 1.282 Å) and are lower than those of N_31_═C_11_, which vary between (1.336 and 1.378 Å). These results also indicate the very high polarization of the C═O bond, which may imply better orbital overlap due to additional p interaction and gives C═O a reinforced bond character on the alumina surface.

### 4.3 Global Molecular Reactivity

Theoretical knowledge of the HOMO and LUMO energy levels of dyes cannot be ignored when studying suitable materials for the adsorption phenomenon as it is a very important factor in determining the intermolecular charge transfer between the donor (adsorbate) and the acceptor (adsorbent).

To study the effect of protonation and deprotonation of the dye by three selected forms that predominated in the pH range on the dye's electronic properties, we calculated the HOMO and LUMO energy levels and their associated descriptors, such as energy gap and electronegativity, for all forms using the B3PW91/LANL2DZ (Table 3). However, based on the analysis of the HOMO and LUMO levels shown in Table 3, we observe that the HOMO energy levels of forms 1 and 2 are lower than the reference level, while form 3 is higher than the reference level. This would suggest that form 3 has a greater capacity to donate electrons for adsorption onto the material. Such a property could be the result of the effect of deprotonation on the oxygen atom O_29_. In contrast to these results, LUMO energy analysis shows that the LUMO energy value of form 3 is higher than that of the reference molecule, while forms 1 and 2 are lower than that of the reference molecule, resulting in a reduced donor effect for these two forms. Furthermore, form 2 has the lowest LUMO energy value, which would theoretically lead to an improved acceptor effect for form 2 compared to that for the other forms.

**TABLE 3:** Conceptual DFT chemical quantum parameters calculated from frontier orbitals for the dye azorubine at B3PW91/LANL2DZ.

	E122	Forme 1(−2)	Forme 2(−1)	Forme 3(−3)
*E*_Total_, eV	−1424.620	−1424.642	−1425.176	−1423.961
*E*_HOMO_, eV	−1.184	−1.243	−3.797	2.258
*E*_LUMO_, eV	1.469	1.160	−2.987	4.834
*I,* eV	1.184	1.243	3.797	−2.258
*A,* eV	−1.469	−1.160	2.987	−4.834
*E*_g_, eV	2.652	2.403	0.810	2.576
*µ*, D	14.776	10.469	13.524	6.027
*χ,* eV	−0.142	0.041	3.392	−3.546
*ɳ,* eV	1.326	1.201	0.405	1.288
*µ,* eV	0.142	−0.041	−3.392	3.546
*σ,* eV^−1^	0.754	0.832	2.470	0.776
*ω,* eV	0.008	0.001	14.206	4.882
*ξ,* eV	130.812	1395.538	0.070	0.205
*ω* ^−^, eV	0.102	0.172	15.953	3.270
*ω* ^+^, eV	0.245	0.130	12.561	6.816
Δ*N,* eV	0.143	0.130	−0.197	0.444

Indeed, when we analyzed the reactivity of these azorubine dye forms, we observed that the lack of electrophilic reactivity (high *E*_LUMO_) in form 3 is compensated by its high nucleophilic reactivity (high *E*_HOMO_). Conversely, in form 2, the deficit in nucleophilic reactivity (low *E*_HOMO_) is compensated by its electrophilic reactivity (low *E*_LUMO_). According to the results discussed above, form 3 would therefore be more reactive than the reference form in the case of an attack governed by the donor effect of these forms. Similarly, form 2 would be more reactive than the reference form in the case of an attack governed by the acceptor effect of these forms.

The energy gap (Δ*E*) values show that the value (0.810 eV) for form 2 is lower than the value (2.403 eV) for form 1, lower than the value (2.576 eV) for form 3, and lower than the reference value (2.652 eV), indicating that form 2 is more reactive than the other forms. Indeed, the lower Δ*E* value indicates that the ability to donate electrons to the surface of Al_2_O_3_ is greater for form 2 (protonated N_31_ and deprotonated O_29_) with a value of 0.810 eV than for the other forms. Form 2 is more likely to interchange its electron clouds with the surrounding environment of the title compounds according to the molecular chemical hardness (*η*) and softness (*S*) values. Form 2 is significantly electrophilic, as evidenced by its higher electrophilicity index value (14.206 eV) compared to that of other forms [33, 34]. Chemical potential (*µ*) is an important factor in understanding the stability and reactivity of a molecule. The negative value of chemical potential is electronegativity. This value determines a molecule's tendency to attract electrons. According to the table, form 2 is more stable than the other forms since it has a lower *µ* value. More negative values for maximum electron charge transfer (Δ*N*) indicate greater electron transfer from the system to the molecule, resulting in increased reactivity, particularly as an electrophile. A more negative Δ*N* is generally associated with a greater capacity to accept electrons in charge‐transfer processes. For form 2 and E122, strong polarity (substantial charge separation) is indicated by a high dipole moment, which frequently increases the molecule's reactivity with polar or ionic species and affects its solubility or boiling point. However, binding strength is just one of several variables that affect reactivity. The E122 molecule and form 3 have negative electron affinity (*A*) and electronegativity (*χ*) values, which show that it is energetically unfavorable to add an additional electron. These molecules are hesitant to take electrons and indicate low electron acceptance because the electron–electron repulsion outweighs the electron‐nucleus attraction [35]. This conclusion, consistent with the molecular energy surface potential (MEP), implies that these chemical compounds are unfavorable for receiving electrons, likely due to a high local electron density.

### 4.4 Density of State (DOS) Analysis

The DOS is performed to investigate the participation of electronic states that retain electrons in a particular energy state throughout the entire molecular system [36]. DOS simulation, executed at the DFT level, uses the GaussSum package to manipulate the charge distribution [37]. The DOS study is an essential tool for characterizing the distribution of energy levels.

In the DOS plots, the blue bands correspond to the acceptor group, the red bands to the donor groups, and the slight blue band represents the overall charge transfer between FMOs. A peak at zero means there are no states at that level, while a higher DOS value indicates a greater contribution (Figure 3). The HOMO–LUMO band gap corresponds to the energy region where no electronic states are present. The calculated band gap values are in good agreement with those obtained from the frontier molecular orbital (FMO) analysis, thereby confirming the consistency of the DOS results with the FMO findings.

**FIGURE 3:**
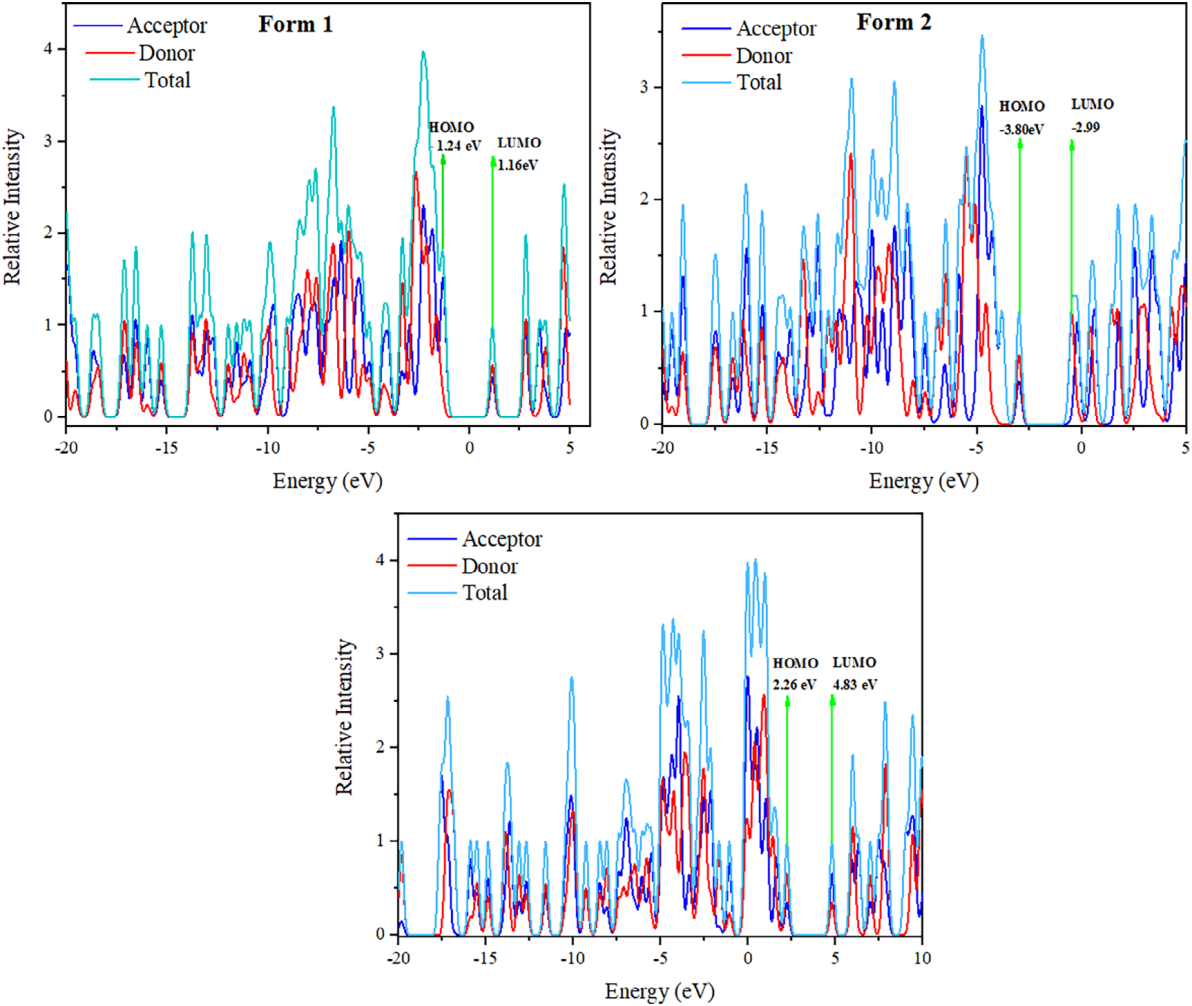
Visual representation of the DOS for the investigated azorubine forms.

### 4.5 Electron Density of HOMO and LUMO Molecular Orbitals and MEP of the Three Forms

Analysis of the chemical reactivity of FMOs, the HOMO, indicates the regions of the molecular system that tend to donate electrons to electrophilic species, while LUMO predicts the regions of the molecular system with a strong tendency to accept electrons from nucleophilic species, and the MEP to understand the absorption and charge transfer processes that are mainly associated with FMOs of all the forms of the dye studied are represented in Figure 4.

**FIGURE 4:**
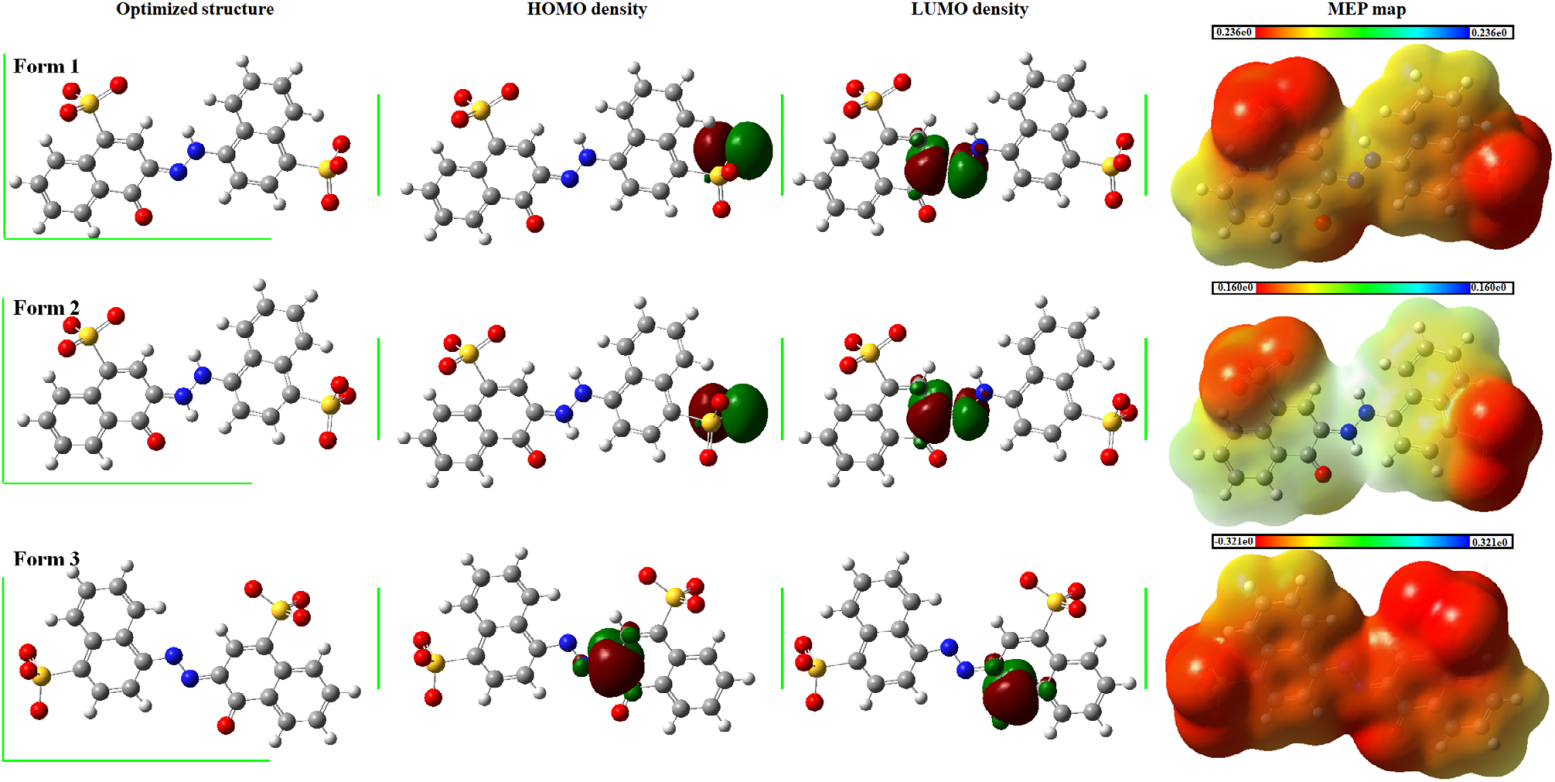
Optimized structures and HOMO and LUMO molecular orbitals and MEP of protonated and deprotonated forms of the dye azorubine calculated by DFT/B3PW91/LANL2DZ.

Figure 4 illustrates the FMO representations for the optimized structures of the azorubine dye forms obtained by the DFT/B3PW91/LANL2DZ method. Forms 1 and 2 have similar FMO electron density distributions. We observe that the electron density of the HOMO of both forms is localized to the sulfuric acid group, while the LUMOs are primarily localized to the naphthylazo region. Furthermore, the electron density of the HOMO for form 3 is localized to the naphthalene region of the molecule, whereas, in the case of the LUMO orbital, the electron density is localized to the same HOMO region.

The electronic delocalization within the studied systems explains the intramolecular charge‐transfer process and, consequently, the efficient adsorption of the azorubine dye onto the Al_2_O_3_ surface. This result is then confirmed by the electrostatic potential of the molecules.

The molecular electrostatic potential generated by electron density is a very useful descriptor in detecting sites for electrophilic and nucleophilic attack reactions, as well as hydrogen bonding interactions [38, 39]. Different colors are used to represent the electrostatic potential map values of a molecule (red, yellow, green, light blue, and blue). Similarly, the MEP surface images of all E122 forms, shown in Figure 4, reveal that regions of high negative charge are observed around the SO_3_^−^ group, which is susceptible to electrophilic attacks. The electron density distribution of the molecules shows the presence of a high negative charge (red color) around oxygen atoms and nitrogen regions, as well as some carbon atoms, for all species studied in aqueous phases. The red and yellow areas represent regions of high electron density, while the blue and light‐blue areas represent regions of low density. In form 1, the electron density is highest around nitrogen (N) and carbon (C) atoms and is significantly higher around sulfonic acid group (─SO_3_H). Conversely, a low electron density is found around hydrogen (H) atoms. The MEP surface also shows a significant distribution of positive charge in form 2 (with a light‐blue color), which becomes relatively prominent in the region around the azo group and some naphthalene ring atoms. Thus, the population analysis based on MEP allows us to consider oxygens more than nitrogens for interaction with the alumina adsorbent. From all these results, we can conclude that the three forms of the E122 dye are more reactive due to the presence of aromatic rings, in addition to heteroatoms, in the process of electron donation and acceptance.

### 4.6 Analyse NCI‐RDG

Figure 5 illustrates the intramolecular interactions occurring in 3D space between the conformers of the azorubine E122 dye and the Al_2_O_3_ surface. NCI analysis, based on the RDG and electron density (*ρ*), was used, as shown in Equation (13) [40].

**FIGURE 5:**
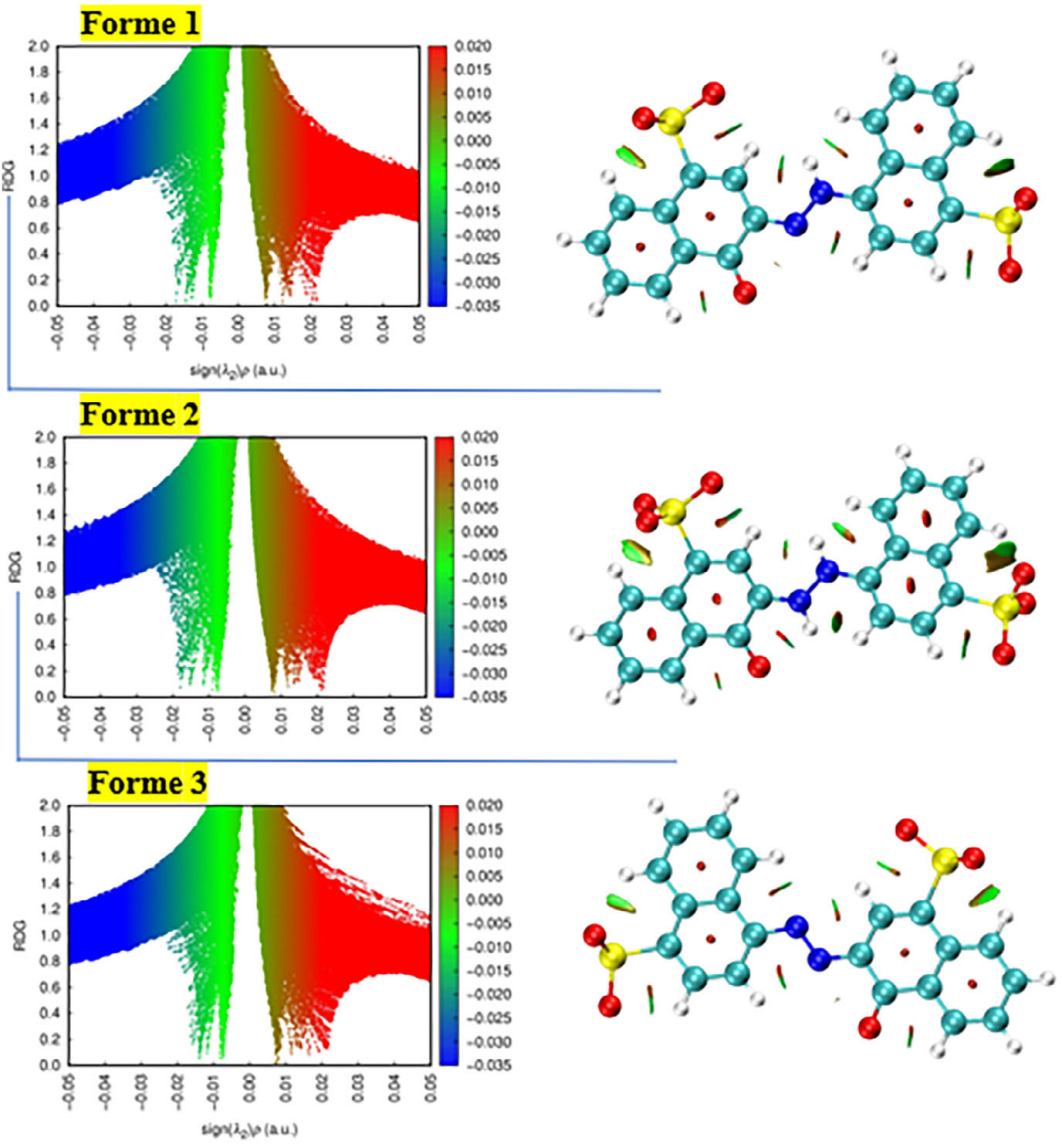
Iso‐surface and NCI pictures for E122.

(13) $$\mathrm{RDG}=\frac{1}{2{\left(3\pi^{2}\right)}^{1/3}}\;\frac{\left|\Delta \rho \right|}{\rho^{4/3}}$$

where Δ*ρ* represents the first derivative of the electron density (*ρ*). The RDG maps (*s* = 0.5 a.u.), correlated with the sign of (*λ* ^2^)*ρ*, allow for the analysis of variations in electron density and provide information on the NCIs that govern molecular behavior by studying the heterogeneity of the electron distribution.

These plots, shown in Figure 5, are bounded by RDG (0.0–2.0) and the sign of (*λ*_2_)*ρ* (−0.05–0.05 u.a.). The sign of *λ*_2_ allows for the qualitative characterization of attractive interactions (*λ*_2_ < 0) and repulsive interactions (*λ*_2_ > 0). The combination sign(*λ*_2_)*ρ* thus makes it possible to qualitatively distinguish the nature and intensity of NCIs. In general, the intensity of NCI decreases as expected with increasing interatomic distance. NCI diagrams feature three distinct regions. However, the figure highlights the most notable regions: the blue region indicates strong interactions created by hydrogen bonding for the parameters (*ρ* > 0, sign(*λ*_2_)*ρ* < 0), the green region represents weak attractive interactions, primarily van der Waals and dispersive in nature (*ρ* ≈ 0, sign(*λ*_2_)*ρ* ≈ 0), and the red region indicates strong steric repulsion (*ρ* > 0, sign(*λ*_2_)*ρ* > 0) [41, 42].

Color intensity reflects a qualitative measure of localized electron density: low RDG values indicate localized NCIs, and the values approach zero at covalent bonds. Figure 5 shows that van der Waals forces play a crucial role in binding the azorubine dye forms (form 1, form 2, and form 3) to the surface of the underlying adsorbent via cooperative π…π, C–H…π, and A…π interactions (A: N, O, S). This suggests that these NCIs contribute to the stabilization of the different forms of the dye (E122 molecule) on the adsorbent surface. NCI/RDG maps confirmed significant weak attractive interactions and localized steric repulsion. Crucially, the three forms of the E122 dye exhibited the strongest attractive van der Waals interactions, which can be described as falling within a range of ≈ (−0.01 to −0.02 a.u.). Thus, the stronger attractive interactions have more negative values in the range of (−0.03 to −0.05 a.u.), corresponding to attractive interactions stronger than van der Waals interactions.

### 4.7 Local Molecular Reactivity

To study the selectivity of the systems investigated, we calculated the natural populations of atoms in the different species: neutral, cationic, and anionic. These populations were used to calculate the Fukui indices (*f*_k_^−^ and *f*_k_^+^), which notably allow us to account for the regioselectivity of the azorubine dye in the three selected forms. Thus, *f*_k_^−^ and *f*_k_^+^, respectively, inform us about the response of the electron distribution following the loss or gain of an electron and consequently about the nucleophilic (high *f*_k_^−^) and electrophilic (high *f*_k_^+^) centers that will undergo electrophilic and nucleophilic attacks, respectively [43].

In Figure 6, we present the most relevant Fukui index values for the azorubine dye. Analysis of the Fukui index values of the azorubine dye in the different forms shows that form 1 has six centers susceptible to nucleophilic attack (electrophilic centers), with high *f*_k_^+^ values: C_9_, C_13_, C_14_, O_29_, N_30_, and N_31_. The results also show that this form also contains eight sites with high *f*_k_^−^ values: C_1_, C_2_, C_7_, C_8_, C_9_, C_11_, C_14_, and N_30_, making these atoms susceptible to electrophilic attack (nucleophilic centers). For form 2, the most notable Fukui index values are for five electrophilic centers with high *f*_k_^+^ values: C_9_, C_13_, O_29_, N_30_, and N_31_, as well as three nucleophilic centers with high *f*_k_^−^ values: O_26_, O_27_, and O_28_. Regarding form 3, the Fukui index values show that this form has five centers susceptible to nucleophilic attacks, with high *f*_k_^+^ values C_8_, C_9_, O_29_, N_30_, and N_31_; thus, it has six centers susceptible to electrophilic attacks, with high *f*_k_^−^ values: C_8_, C_9_, C_11_, C_14_, O_29_, and N_30_.

**FIGURE 6:**
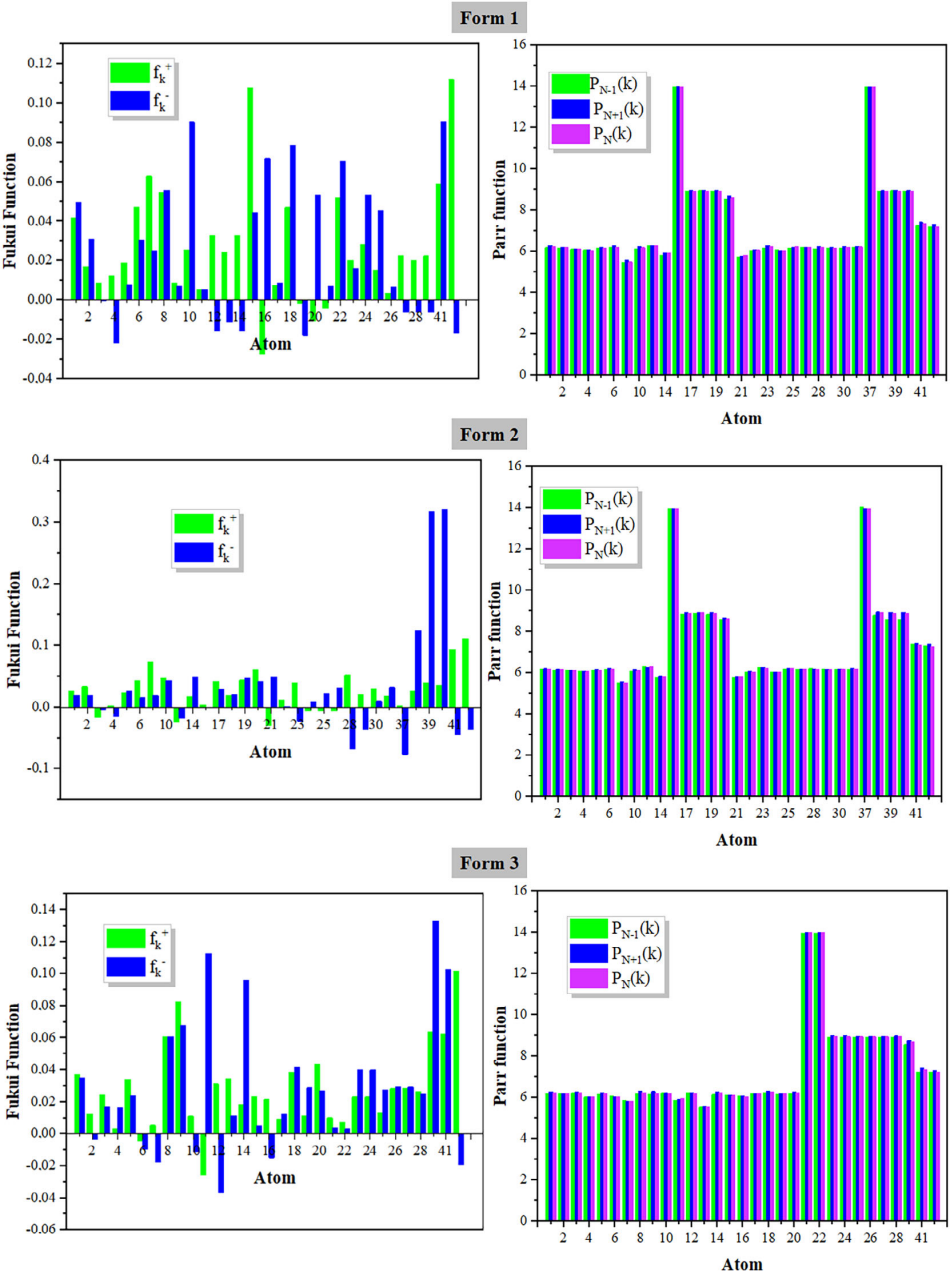
Fukui functions (*f*_k_^+^: green, *f*_k_^−^: blue) of the HOMO and LUMO molecular orbitals of the three azorubine forms calculated at the B3PW91/LANL2DZ theory level.

The Fukui index values in form 1 show that the carbon atoms C_9_ and C_14_ and the nitrogen atom N_30_ possess dual electrophilic and nucleophilic character. Thus, in form 3, the azorubine molecule has four sites with dual electrophilic and nucleophilic character: C_8_, C_9_, O_29_, and N_30_. However, it is important to note that the Fukui index values *f*_k_^−^ calculated for the oxygen atoms in form 2 are much higher than those for the other atoms, which are ≈0.317 and 0.322 for the oxygen atoms (O_27_ and O_28_), respectively. These high Fukui index values for the oxygen atoms indicate a greater predisposition for adsorption of these atoms onto the metal surface compared to the other atoms. It is also noted that the O_27_ and O_28_ sites exhibit a fairly significant electron‐donating characteristic in form 2, resulting in a greater excess of electrons at O_27_ and O_28_, making form 2 more reactive than the other forms.

### 4.8 NBO Analysis

The study of charge‐transfer processes using NBO allows us to explain the type of intramolecular and intermolecular bonding, as well as the properties of a given molecule [44]. The displacement of π electrons within the studied molecule from a given Lewis valence orbital (occupied orbital) follows a decrease in occupancy due to the electron density, which is delocalized to another region of the molecule (unoccupied orbital). Furthermore, this method allows us to study the nature of the charge‐transfer process during the adsorption of the azorubine dye onto the surface of the Al_2_O_3_ substrate. The results obtained for the protonated and deprotonated forms of azorubine, calculated at the DFT/B3PW91/LANL2DZ level, are shown in Table 4.

**TABLE 4:** NBO analysis of the three forms of azorubine using B3PW91/LANL2DZ. *E* ^2^ denotes the energy of hyperconjugative interactions.

**Donor (*i*)**	**Acceptor (*j*)**	***E* ^2^, kcal/mol**			**Donor (*i*)**	**Acceptor (*j*)**	***E* ^2^, kcal/mol**		
		Form 1	Form 2	Form 3			Form 1	Form 2	Form 3
π(C_14_–C_12_)	π*(C_11_–N_31_)	—	39.41	—	σ*C_13_	BD*C_15_–C_16_	—	—	49.59
BD(C_15_–C_16_)	π*(C_18_–C_17_)	22.20	20.77	24.07	nO_29_	σ*C_13_	—	—	223.00
BD(C_15_–C_16_)	π*(C_13_–O_29_)	23.62	31.35	—	BD*C_13_–O_29_	BD*C_15_–C_16_	67.38	51.18	—
BD(C_15_–C_16_)	σ*C_13_	—	—	43.10	BD*C_14_–S_21_	RY*S_21_	21.12	24.38	—
BD*C_15_–C_16_	BD*C_19_–C_20_	—	281.70	—	BD*C_11_–N_31_	BD*C_13_–O_29_	230.64	107.06	—
BD*C_15_–C_16_	BD*C_12_–C_14_	—	—	204.94	BD*C_11_–N_31_	BD*C_14_–C_12_	36.89	36.56	—
π(C_19_–C_20_)	π*(C_18_–C_17_)	20.41	20.50	—	BD*S_21_–O_23_	BD*C_14_–S_21_	43.39	84.60	20.00
π(C_19_–C_20_)	π*(C_15_–C_16_)	20.95	23.07	259.62	BD*S_21_–O_24_	BD*C_14_–S_21_	40.23	75.34	20.00
BD*C_18_–C_17_	BD*C_19_–C_20_	—	217.35	—	BD*S_21_–O_25_	BD*C_14_–S_21_	43.38	85.39	22.28
BD(C_17_–C_18_)	BD*(C_15_–C_16_)	20.19	—	162.18	BD*C_7_–C_8_	BD*C_10_–C_9_	176.67	73.05	—
BD(C_4_–C_6_)	π*(C_7_–C_8_)	20.15	22.60	—	BD*C_7_–C_8_	BD*C_4_–C_6_	—	129.11	—
BD(C_10_–C_9_)	π*(C_4_–C_6_)	20.13	—	—	BD*C_4_–C_6_	BD*C_10_–C_9_	230.42	247.10	
BD(C_10_–C_9_)	BD*(C_7_–C_8_)	21.82	—	—	BD*C_4_–C_6_	BD*C_5_–C_3_	267.12	137.94	—
BD(C_5_–C_3_)	BD*(C_2_–C_1_)	21.87	22.83	—	BD*C_2_–C_1_	BD*C_5_–C_3_	118.26	89.00	—
*n* N_30_	BD*(C_11_–N_31_)	45.14	29.23	—	BD*C_9_–S_22_	RY*S_22_	20.72	23.19	—
*n* N_30_	BD*(C_7_–C_8_)	32.30	28.52	—	BD*S_22_–O_26_	BD*C_9_– S_22_	36.94	58.88	22.03
*σ* C_11_	BD*(C_14_–C_12_)	—	—	67.89	BD*S_22_–O_27_	BD*C_9_–S_22_	37.02	59.22	22.03
*σ* C_11_	BD*(N_30_–N_31_)	—	—	163.93	BD*S_22_–O_28_	BD*C_9_–S_22_	36.95	58.88	23.66

In this context, the strongest interactions between occupied and unoccupied natural orbitals are represented by the largest *E* ^2^ values (stabilization energy). The main possible interactions found in organic systems are (n → π*), (π → π*), and (n → σ*). Furthermore, the highest interaction energies in the azorubine dye reflect conjugation in these studied systems. Table 4 reveals that the highest intramolecular charge transfers of form 1 range from (π*C_11_–N_31_ to π*C_13_–O_29_), (π*C_4_–C_6_ to π*C_10_–C_9_), (π*C_4_–C_6_ to π*C_5_–C_3_), and (π*C_2_–C_1_ to π*C_5_–C_3_) with stabilization energies of the order of 230.64, 230.42, 267.12, and 118.26 kcal/mol, respectively. The stabilization energies for form 2 are roughly 281.70, 217.35, 107.06, 247.10, 137.94, and 89.00 kcal/mol for the following interactions: (π*C_15_–C_16_ to π*C_19_–C_20_), (π*C_18_–C_17_ to π*C_19_–C_20_), (π*C_4_–C_6_ to π*C_13_–O_29_), and (π*C_2_–C_1_ to π*C_5_–C_3_). In form 3, the interactions of π*C_15_–C_16_) with π*(C_12_–C_14_), π(C_19_–C_20_) with π*(C_15_–C_16_), π(C_17_–C_18_) with π*(C_15_–C_16_), σ(C_11_) with π*(N_30_–N_31_), and n(O_29_) with σ*(C_13_) give stabilization energies of 204.94, 259.62, 162.18, 163.93, and 223.00 kcal/mol, respectively. Significant charge transfers between antibonding molecular orbitals (π*) and (π*) suggest significant reactivity and conjugation in the examined compounds. These (π*) orbitals, positioned at the highest energy levels, are less stable, therefore enabling charge transfers.

### 4.9 Numerical Simulation

Numerical simulation methods at the atomic scale that allow us to ascertain the physical characteristics of a system of interacting particles are found in the discipline of statistical physics. These include off‐network techniques that relax atomic positions (MD) to describe the equilibrium structures of a system and its evolution and rigid network techniques (mean field, MC) when only chemical order is relevant (between two species, or even one species and vacancies in the case of adsorption) [23, 24, 45, 46]. The most advantageous configurations for the adsorbed species at low‐energy adsorption locations on the surface were successfully predicted by the MD approach. The best configurations for the system under investigation are shown in Figure 7a. According to simulations, Figure 7a depicts dye molecules moving parallel to a surface, demonstrating a surface diffusion or lateral transport phenomenon in which the particles interact strongly with the substrate and move primarily in the surface plane rather than crossing into the volume. This phenomenon is frequently caused by adhesion forces, particular concentration gradients, or surface characteristics. Dye adsorption on Al_2_O_3_ occurs via interactions involving the unshared electron pairs of heteroatoms (such as nitrogen and oxygen) as well as π electrons from aromatic rings, which interact with the vacant p orbitals of surface Al atoms, forming strong physical/chemical bonds. This makes Al_2_O_3_ an excellent adsorbent for removing dyes from water. Figure 7b illustrates the dye's density field distribution on the Al_2_O_3_ surface (113). The distribution of the density field of adsorption of a molecule on an adsorbent explains how the electrons of the adsorbate (the molecule) redistribute and interact with the electrons of the adsorbent (the surface) during adhesion, forming regions of higher or lower electron density (hydrogen bonds, Van der Waals, and polar centers). This information is essential for comprehending interaction energy, bond strength, and particular adsorption sites, which affect the storage capacity or catalysis. This data indicates that the creation of a 3D dense barrier layer is likely the cause of the significant adsorption.

**FIGURE 7:**
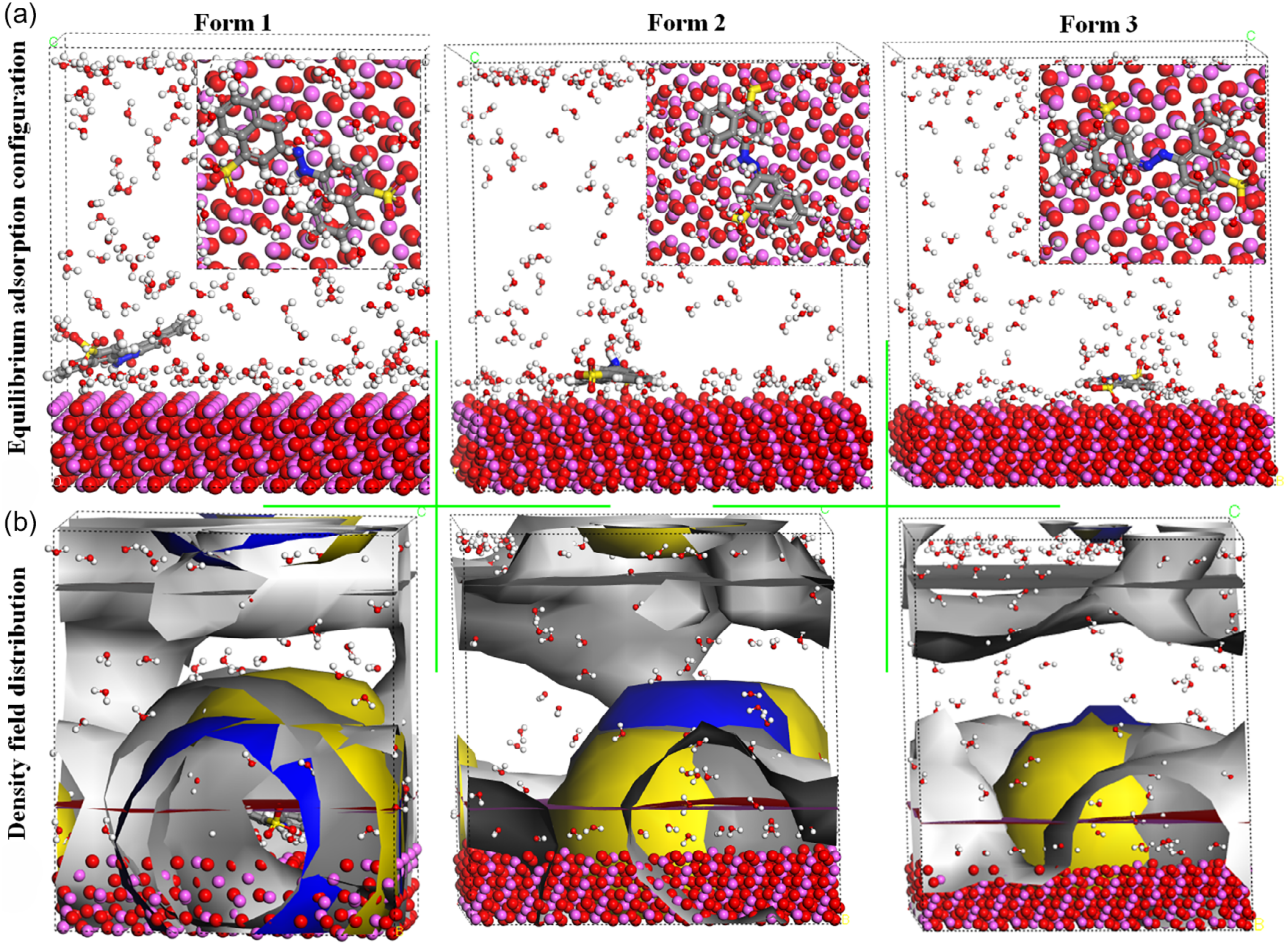
(a) Equilibrium adsorption configuration of studied molecules on the Al_2_O_3_(113) surface and (b) density field distribution.

#### 4.9.1 RDF

Based on the analysis of MD trajectories, the RDF was utilized to distinguish between physical and chemical interactions between the adsorbate molecule and the adsorbent surface. Generally, a peak position below 3.5 Å indicates chemisorption (chemical interaction), characterized by strong bonds such as covalent or coordination bonds. Conversely, peak distances above 3.5 Å are indicative of physisorption (physical interaction), governed by weaker forces such as van der Waals interactions and hydrogen bonding [47, 48, 49, 50, 51]. The radial distance r corresponding to each first conspicuous peak is summarized in Figure 8, which also displays the RDF curves of the various oxygen and nitrogen atoms of the molecules with the closest atoms on the Al_2_O_3_ (113) surface. The findings indicate that the Al_2_O_3_ (113) surface and the oxygen and nitrogen atoms of the three molecules interact attractively. Since all of the radial distances are shorter than 3.5 Å, these interactions are chemical in nature and demonstrate the atoms’ reactivity as well as their important involvement in the adsorption process by forming hydrogen bonds with the adsorption surface. These findings align with the previously mentioned quantum deductions. Additionally, this figure shows that weak physical interactions typical of van der Waals forces (London, Keesom, and Debye dispersion forces) are represented by the peaks at great distances (*r* > 3.5 Å). In contrast to chemical adsorption (chemisorption), in which the atoms interact weakly with the surface and contribute nothing to its reactivity or structure, these weak forces, such as Van der Waals forces, confirm physical adsorption (physisorption).

**FIGURE 8:**
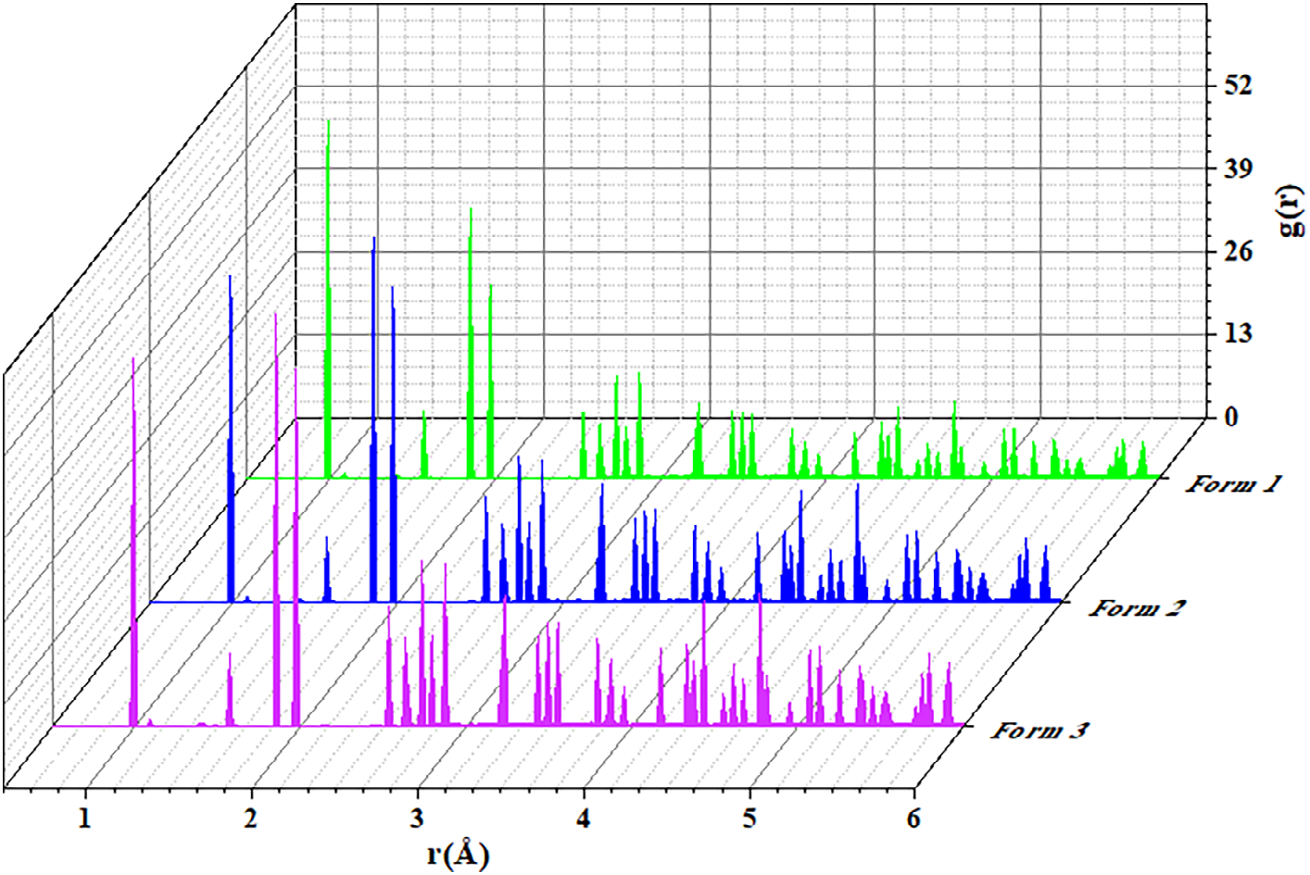
The RDF analysis of the interaction between studied molecules and Al_2_O_3_ adsorbent.

#### 4.9.2 Dynamic Descriptors

The adsorption energy (*E*_ads_), which is the energy transferred during a molecule's attachment to a surface, is the difference in energy between the adsorbed system and its separated components. A favorable adsorption (exothermic), whether by chemical (strong chemisorption) or physical (weak physisorption), is indicated by negative values [49, 52, 53, 54]. Furthermore, the rigid adsorption energy (RAE), which represents the energy required to release the adsorbate without deformation, and the deformation energy (*E*_def_), which corresponds to the alteration of the adsorbent's structure during the interaction, add up to the *E*_ads_ in molecular systems. To comprehend the energy contributions made during a molecule's adsorption on a surface, this breakdown is helpful [24, 27]. The energy needed to extract a single adsorbate molecule from the adsorbent's surface is represented by the *d* *E*_ads_/*d*Ni. In Table 5, the different energy descriptors are computed and are displayed. Higher negative Eads values indicate stronger and more stable adsorption because they mean more energy is released during binding, as in chemisorption, compared to physisorption. For Form 2, a more negative *E*_ads_ (−917.107 kcal/mol) reveals stronger interaction forces, making the adsorbate (Form 2) more difficult to desorb. Form 3 has a lower adsorption energy than forms 1 and 2 in an aqueous solution, according to the Eads data, indicating a weaker contact between form 3 and the surface. This indicates that in an aqueous medium, it forms 2 bonds to the surface more firmly. This information may be essential for identifying the most reactive adsorbate with the surface adsorbent. However, the absolute values of *E*_def_ show the same pattern. Form 3 < Form 1 < Form 2 is the order in which the energy increases. This suggests that before and after the adsorption system was optimized, Form 2 had a greater propensity to interact and adsorb on the Al_2_O_3_ (113) surface. The structural rearrangement during relaxation has made each molecule and the Al_2_O_3_ (113) surface less stable, as indicated by the negative values of *E*_def_ being lower than those of RAE [55, 56]. However, H_2_O molecules cannot compete with chemical compounds for adsorption on the chosen surface because the adsorption energy distribution of the chemical compounds is different and larger (in absolute value) than that of the H_2_O molecule. Additionally, a stronger contact between the dye molecule and the adsorbent surface is indicated by a greater negative *dE*_ads_/*d*Ni value (Figure 9). The outcomes demonstrate that Form 2 and the surface interact more strongly than other forms.

**TABLE 5:** Outputs and descriptors calculated via MD simulation for the adsorption of the forms of dyes studied on the Al_2_O_3_ surface in aqueous solution (kcal/mol).

	*E*_tot_	*E*_ads_	*E*_RA_	*E*_Def_	*d* *E*_ads_/*dN*_dye_	*dE*_ads_/*d*Ni_H__2__O_
Form 1 – Al_2_O_3_	−257.554	−861.553	−498.428	−363.125	−125.967	−1.587
Form 2 – Al_2_O_3_	−256.305	−917.107	−495.369	−421.737	−193.402	−1.489
Form 3 – Al_2_O_3_	−211.986	−806.286	−444.569	−361.717	−130.731	−1.485

**FIGURE 9:**
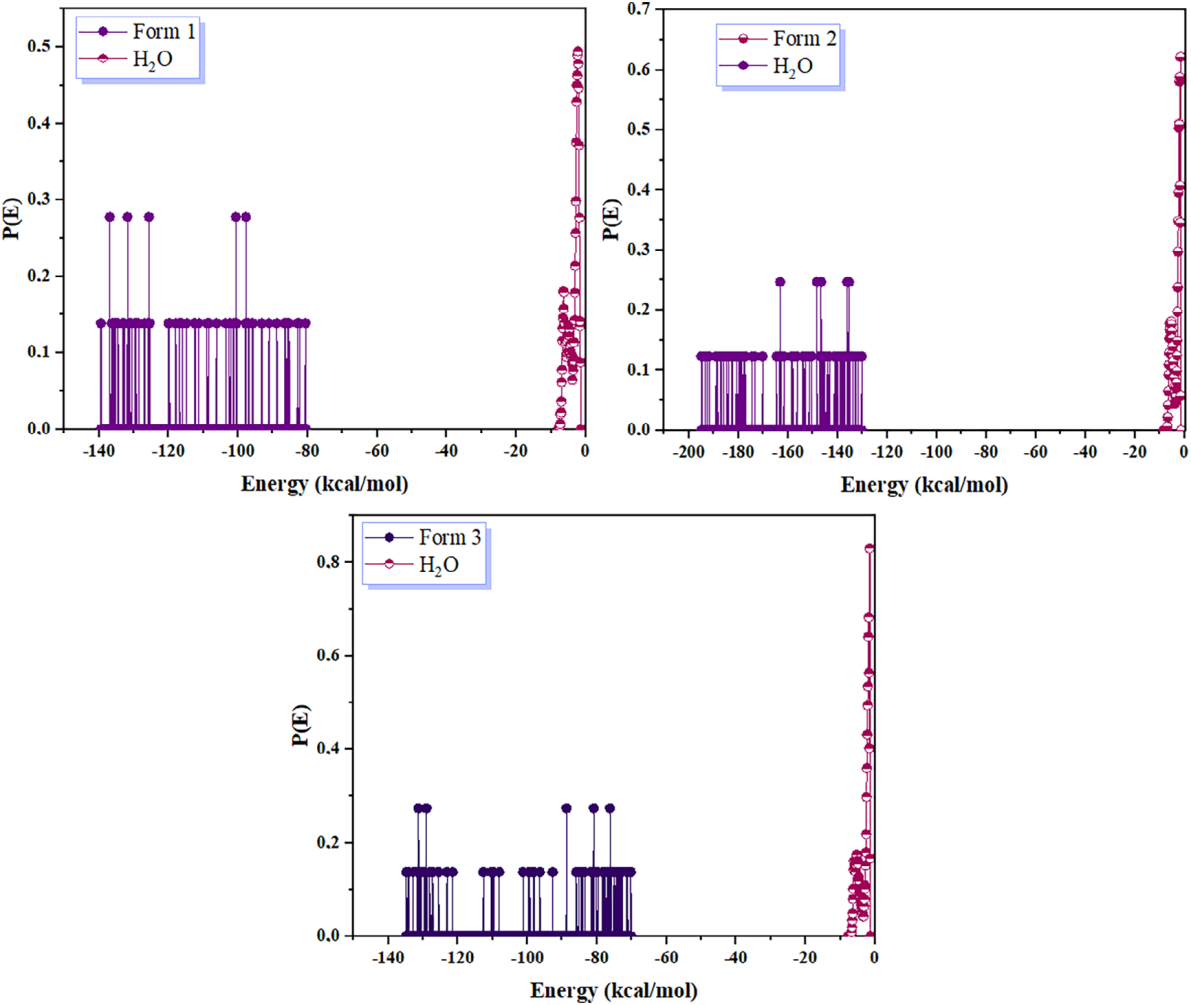
Energy distribution of the chemical compounds on Al_2_O_3_ adsorbent.

#### 4.9.3 Adsorption Mechanism of Dye (113) Al_2_O_3_ Surface

Theoretical simulation, particularly through molecular modeling and quantum mechanics techniques, enables a thorough examination of the interactions (van der Waals forces, hydrogen bonds, electrostatic interactions, etc.). between the dye molecule and the adsorbent surface. This helps comprehend the basic mechanisms of adsorption (chemical vs. physical), the strength of attraction, the orientation of molecules, and the characteristics of active sites, providing important information to optimize decontamination processes. According to the results obtained in this study, the dye exhibits high reactivity and a strong affinity for the (113) surface of the Al_2_O_3_. This affinity can be explained by the involvement of donor–acceptor interactions, such as (i) the n‐π interaction between the nonbonding doublet of heteroatoms on the surface and the π electron of the aromatic rings of the dye; (ii) the hydrogen bonds dipole–dipole between the heteroatoms and oxygen atoms of the surface; and (iii) Yoshida hydrogen bonds between the OH groups of the surface and the aromatic rings of the dye. Furthermore, it should be noted that the Van der Waals interactions can be very important in the adsorption process of the studied substances. The presence of hydroxy sites (Al–OH) on the surface of alumina can be determined by pH. The attraction of positively (cationic) or negatively (anionic) charged dyes can be influenced by these sites, whether they are acidic or basic groups. It is essential to take into account the zero charge point (ZCP) of alumina: if the pH is lower than ZCP, the surface is positively charged. When the pH is higher than that of PCZ, the surface undergoes a negative charge. The mechanism of the adsorption process is schematically represented in Figure 10.

**FIGURE 10:**
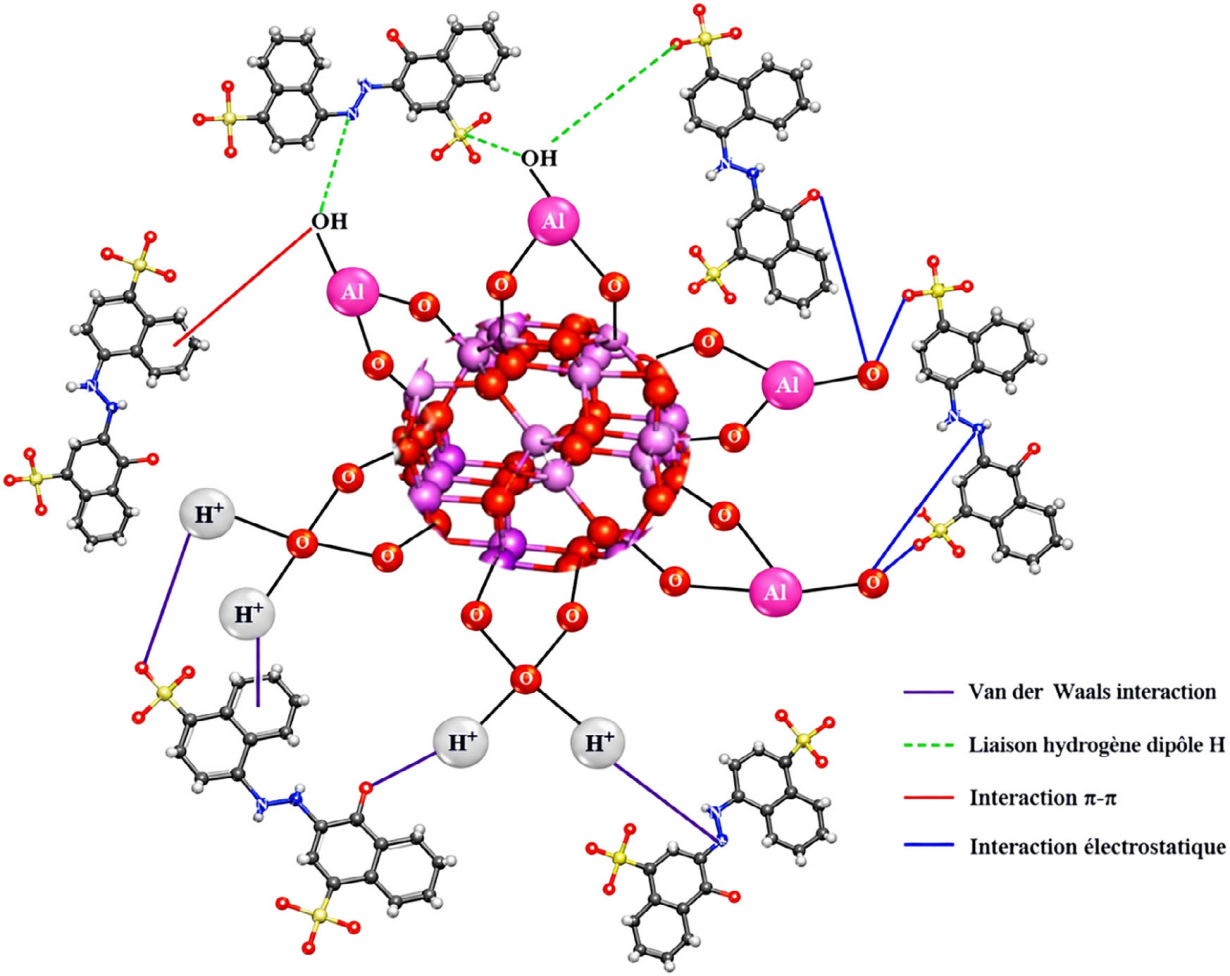
Suggested adsorption mechanism of dye on Al_2_O_3_ surface.

## 5 Conclusion

This study provides new molecular‐level insights into the adsorption of the azo dye azorubine (E122) on the (113) surface of Al_2_O_3_. The results demonstrate that the adsorption behavior depends on the molecular form of the dye, with forms 2 and 3 adopting favorable parallel configurations on the alumina surface. The negative adsorption energies indicate that the adsorption process is energetically favorable, while form 2 exhibits the strongest interaction with the Al_2_O_3_ surface among the investigated forms.

The main scientific contribution of this work is the identification of the most favorable molecular forms and adsorption configurations of E122 on an alumina surface. These findings improve the molecular understanding of the interactions governing azo‐dye adsorption and provide insights into how the molecular state of the pollutant can influence its adsorption behavior. This contributes to the development of a more rational approach for understanding and optimizing the adsorption of azo dyes on inorganic adsorbent materials.

From a practical perspective, the results indicate that Al_2_O_3_ has the potential for the removal of azorubine from aqueous media. The molecular‐level information obtained in this study can be useful for guiding the development and optimization of alumina‐based adsorbents for dye‐contaminated water treatment. However, further experimental investigations are required to validate the predicted adsorption behavior and evaluate the performance of the material under realistic aqueous conditions.

## Author Contributions

**Anouar Ameziane El Hassani**: conceptualization, data curation, formal analysis, methodology, writing – original draft, writing – review, and editing. **Malika Khnifira**: conceptualization, data curation, writing – review and editing. **Issam Ameziane El Hassani**: conceptualization, data curation, writing – review and editing. **Mohamed Adadi**: conceptualization, data curation, writing – review, and editing. **Mohammed ALSaeedy**: validation, resources. **Adil Touimi Benjelloun**: conceptualization, data curation, writing – review, and editing. **Mohamed Abdennouri**: conceptualization, data curation, writing – review, and editing.

## Funding

The authors have nothing to report.

## Ethics Statement

The authors have nothing to report.

## Consent

The authors have nothing to report.

## Conflicts of Interest

The authors declare no conflicts of interest.

## Data Availability

No data was used for the research described in the article.
